# RGB-Detector: A Smart, Low-Cost Device for Reading RGB Indexes of Microfluidic Paper-Based Analytical Devices

**DOI:** 10.3390/mi13101585

**Published:** 2022-09-23

**Authors:** Bianca Maria Pazzi, Dario Pistoia, Giancarla Alberti

**Affiliations:** Department of Chemistry, University of Pavia, Via Taramelli 12, 27100 Pavia, Italy

**Keywords:** RGB-detector, Arduino-based device, low-cost sensor, microfluidic paper-based analytical devices, color analysis

## Abstract

A user-friendly, low-cost detector able to read the RGB indexes of microfluidic paper-based analytical devices (µPADs) was developed. The RGB-detector was built with 3D printing using PLA+ and reused Li-ion batteries. It is Arduino-based, which provides an easy interface between the sensor TCS3200, which reads the quadratic wave of the times corresponding to the RGB numbers, the Arduino itself, whose software translates the times into RGB values, and the touchscreen display, NX3224T028, which shows the results. This detector permits multi-sample analysis since it has a sample holder that can keep up to six µPADs simultaneously and rotate after the display’s request. This work shows how the readings of the RGB indexes by the proposed RGB-detector implement the measurements’ reproducibility. As a proof-of-concept, the RGB-detector application to a green array of µPADs for pH measurement coupled with chemometric analysis allowed us to achieve good results in terms of precision and agreement with the pH values measured by a classical pH-meter.

## 1. Introduction

Color in chemistry is a crucial aspect. Color change in a solution could indicate the occurrence of a chemical reaction; moreover, color intensity is related to the concentration of an analyte that absorbs light in the visible range of the spectrum. Colorimetric techniques have been developed and widely used in routine qualitative and quantitative analyses [1].

UV-vis spectrophotometers and colorimeters are the usual analytical instruments used for colorimetric measurements. In colorimetric analyses, the acquisition of the signal is a crucial step. Sensing and sensitivity depend on the detection methods and the type of detector employed. Colorimetric analyses are based on the interaction of visible radiation with a colored solution or solid phase. Absorbance and % transmittance are the most employed detection modes in UV-vis spectroscopy and colorimetry. Absorbance is correlated to the intensity of monochromatic radiation absorbed by the analyte. Conversely, the transmittance is based on the intensity of monochromatic radiation not absorbed by an analyte. A reflectance detection approach is instead employed for colored solid substrates such as Paper-based Analytical Devices (PADs); it is based on the measure of the light reflected from the solid surface [1].

Although spectrophotometers are the gold standard techniques in laboratories worldwide, middle- and low-income countries cannot afford these expensive instruments and do not have the necessary infrastructure and expertise. Alternatively, smartphones, scanners or digital cameras were proposed as low-cost detectors for colorimetric assays performed by test strips or paper-based devices [2]. These tools return the color information by using a color space, i.e., a visualization that describes the color spectrum as a multidimensional model, including RGB (Red, Green and Blue), associated with smartphones screens and digital cameras [3]. Although attractive, these devices show some drawbacks, such as the sensitivity variation among different smartphones and the need for image processing by a computer or a mobile application to acquire RGB values, making real-time monitoring more time consuming [4,5,6].

The development of integrated low-cost digital platforms, for example, Arduino [7], Raspberry PI [8] or Mbed OS [9], is increasing for prototyping cheap instruments such as colorimetric detectors [3,10,11,12,13]. Arduino is renowned for the prototype development of colorimetric detectors, thanks to its ease of use, simple programming, and reasonable price [3,14,15,16,17,18,19,20,21,22,23,24,25].

In this scenario, an Arduino-based, user-friendly, low-cost detector able to read the RGB indexes of microfluidic paper-based analytical devices (µPADs) is developed. Arduino UNO is selected for its hardware versatility and compatibility; indeed, given an electronic scheme, it can be used as a motor controller. The device, named the RGB-detector, has a casing that is 3D printed with polylactic acid plus (PLA+) and is powered by reused Li-ion batteries. The TCS3200 Color Sensor is employed; it is a color detector, including an RGB sensor chip and four white LEDs. A touchscreen display NX3224T028 permits the immediate reading of the RGB values.

The capability of the RGB-detector to quantitatively measure the color of microfluidic paper-based analytical devices (µPADs) is demonstrated. In particular, as a proof-of-concept, it is applied to an array of green µPADs for pH measurements; good results in terms of precision and agreement with pH values measured by a classical pH-meter are achieved. The good performances obtained makes the RGB-detector promising for application to other microfluidic devices based on different substrates such as fused silica, polydimethylsiloxane, polystyrene (PS), poly(methyl methacrylate) (PMMA), and polyvinylalcohol (PVA and polycarbonate (PC) [26,27]. Moreover, the possibility of 3D printing interchangeable sample-holders for microfluidic devices of different shapes and dimensions is an added benefit for expanding the applicability of the RGB-detector prototype realized here.

## 2. Materials and Methods

### 2.1. Materials for Building the RGB-Detector

The Arduino Uno microprocessor (Rev3 Arduino) was employed to realize the RGB-detector. The RGB TCS3200 Color Sensor (Arceli, Vicenza, Italy) was used. A 2.8” Basic Series HMI Touch Display NX3224T028 (Nextion, Shenzhen, China) permitted the immediate reading of the RGB values. A digital micro servomotor MG996R (AZDelivery, Deggendorf, Germany) was used to manage the correct positioning of the samples in the holder. PCB BMS Li-Ion board (Aideepen, Shenzhen, China) and 3,6V (3s) Li-Ion batteries (Samsung Electronics Italia S.p.A, Milano, Italy) were employed. Polylactic acid plus (PLA+, Sunlu, China) was used to 3D print the detector case and all other components.

### 2.2. Materials for RGB-Detector Calibration and Application

#### 2.2.1. RGB-Detector Calibration

Colored cards (148 × 210 mm—DIN-A5, Procket, Berlin, Germany) were cut into squares of 2 cm per side. The RGB indexes of each square, obtained by a comparison performed with the 216 Web Safe Colour Chart (https://www.greywhitebalancecolourcard.co.uk/ (accessed on 29 August 2022)), were used to calibrate the detector experimentally.

#### 2.2.2. RGB-Detector Application: Green µPADs Array for pH Measurements

A sheet of filter paper was cut into squares of 2 cm per side with a letter opener to keep the area constant. Each square is placed on a clean, flat surface, drop-coated with 0.2 mL of natural dye extracts from red cabbage (*Brassica oleracea*, RC) and butterfly pea flower (*Clitoria ternatea*, BPF) alone or in a mixture. Then, the prepared PADs were allowed to air dry. The following five extracts were employed: 100% RC, 100% BPF, a mixture of 25% RC-75% BPF, a mixture of 50% RC-50% BPF, and a mixture of 75% RC-25% BPF. Thus, an array of 5 kinds of µPADs was prepared. Each µPAD was then made to interact with 5 mL of solutions at different pH values (from 1 to 13) or sample solutions for some seconds to wet the paper. Then, the µPAD was removed from the solution, left to dry for 5 min on a clean, flat surface, and inserted in the RGB-detector’s sample holder.

The pH of each solution was previously measured with a pH-meter and used as a reference value for the chemometric data treatment.

Solutions at different pH values (ranging from 1 to 13) were prepared by titrating orthophosphoric acid with standardized NaOH. The tap water sample was obtained from the drinking water supply of Pavia (Italy). The sample was collected after flushing cold water for 20 min from the sink of the laboratory (Chemistry Department, University of Pavia, Pavia, Italy). Ammonia cleaner (S.a.i.soc.alcoli Industriali Sas, Roccabianca, Italy), Tropical Aloe Vera drink (Eurofood S.p.A., Corsico, Italy), Schweppes tonic water (Schweppes International Limited, Milano, Italy), Sprite (Coca-Cola S.r.L., Sesto San Giovanni, Milano, Italy), and white wine vinegar “Gaia” (Formec Biffi S.p.A., San Rocco al Porto, Italy) were purchased in a local supermarket (Pavia, Italy).

### 2.3. Chemometric Data Treatment

Chemometric tools are widely applied when developing sensors to obtain analytical information from the responses of chemical devices and arrays. Chemometric tools were applied here to analyze the RGB dataset. A multi-techniques approach was used, combining the following two unsupervised techniques: a Principal Component Analysis (PCA) and Three-Way Principal Component Analysis (3WPCA), with the supervised partial least square regression (PLS) since it has proven to be a very informative data treatment method in terms of predictive performances and the model’s robustness [28,29,30,31].

All the tools applied are extensively reviewed in the literature, so their theoretical background will not be discussed here [32,33,34,35].

3WPCA was selected since it considers the tri-dimensional nature of a dataset, which is believed to be a parallelepiped of a size of *O* × *V* × *C* (conventionally termed Objects, Variables and Conditions). Thus, the information relating to RGB indexes (Variables), the five types of green µPADs (Objects) and the solution pHs (Conditions) are separated, thereby facilitating a clear interpretation of the information present in the dataset.

PCA and 3WPCA were applied to the entire data set. From the resulting PCA’s score plot, the following three pH subintervals are highlighted: pHs from 1 to 4 (acid), pH from 5 to 8 (neutral), and pHs from 9 to 13 (alkaline). Then, the PLS tool was applied separately for each pH subinterval, developing a tailored model correlating the RGB indexes of the PADs sensors with the pH values.

The training set required to build the PLS models included three replicates of each solution; thus, the input matrix had 15 columns (3 RGB indexes per 5 µPADs) and, respectively, 12 lines (4 solutions per 3 replicates) for the interval from pH 1 to 4 and for that from pH 5 to 8, while 15 lines (5 solutions per 3 replicates) were used for the interval from pH 9 to 13. The test set used to validate the PLS models comprised three replicates of each sample solution; so, the test input matrix had 15 columns (3 RGB indexes per 5 µPADs) and, respectively, 12 lines (4 solutions per 3 replicates) for samples with a pH from 1 to 4, while 3 lines (1 solution per 3 replicates) for samples with a pH from 5 to 8 and 3 lines (1 solution per 3 replicates) for samples with a pH from 9 to 13.

## 3. Results

### 3.1. Design of the RGB-Detector

The RGB-detector was developed to measure the RGB indexes of µPADs by avoiding variability due to different types of cameras or brightness of the environment and simplifying the readings.

Design constraints were required to develop a functional and efficient device: the maximum overall dimensions must be contained as the device needs to be portable, the components must be easy to find, assemble, and restore, and it has to be user-friendly, low-cost, and environmentally sustainable. The RGB-detector also needs only one sensor for the RGB reading, a multi-analysis possibility, and standardization of measurement. The functioning logic must be easy to understand, reliable, and durable, providing repeatable readings in an enclosed setting with well-defined lights and sample placement.

The hardware must control the communication between itself, the color sensor, the display, the servo motor, and the batteries. The chosen hardware was Arduino Uno, which is the most suitable given its accessibility and low cost.

The Li-ion rechargeable batteries powered Arduino, the display, and the servo motor. The sensor was connected with the hardware, which received both power and commands to measure RGB indexes, which were later sent to Arduino. From Arduino, RGB indexes were sent to the display, which showed the final results and allowed us to control the position of the desired sample to be analyzed.

### 3.2. RGB-Detector Realization

It was therefore decided to use Arduino as the hardware platform thanks to its ease of use, simple programming, and reasonable price.

A TCS3200 color sensor was selected since it can be controlled simply with Arduino (through digital pins, and the output is provided to the Arduino board through a square wave), which works in the visible spectrum. Through a series of LEDs, it is possible to standardize the incident light to the samples to be analyzed to standardize the sampling process.

The use of an easily programmable touchscreen display was also practical. It allowed us to control sample positioning inside the holder and the immediate display of RGB indexes. We selected the 2.8” Basic Series HMI Touch Display NX3224T028, Nextion, for the simplicity of communication with Arduino and its programming system.

Since a portable instrument was developed, it was equipped with a 12V lithium-ion (Li-ion) battery (3s with a capacity of 2000 mAh) managed by a BMS (Battery Management System) that guarantees the correct discharge and charge of each cell, and that protects the entire electrical circuit from overcurrent. In addition, the instrument has been arranged with the possibility of powering the instrument itself with an external power supply that can simultaneously recharge the battery.

A servomotor was employed to manage the correct positioning of the samples in the black reading holder. A simple model servomotor was selected to obtain a rotation between 0° and 180°, and thanks to a pair of gears designed ad hoc with a ratio of 2:1, it was possible to convert the rotation from 180° to 360°.

The case and all other components were made with 3D printing (FDM) techniques by the deposition of molten material. The material used was PLA+, thanks to its good mechanical characteristics and resistance to external chemical agents.

The main components for which we had to start from scratch for the design were the main box, the cover (upper and display cover), the darkroom panel, the gears, and the sample holder. After the various design constraints were considered for each of them, we moved on to their 3D modeling using CAD software. This step was fundamental for rapid implementation and the feasibility study. All the dimensions and the free space available for the electrical and electronic parts have been optimally evaluated.

For the case, i.e., the container of the entire instrumentation, the maximum dimensions were defined at the preliminary stage and compatible with the number of samples it should have contained. Side openings have also been provided to facilitate the connection to the Arduino board to load some software updates quickly.

The µPAD-holder must ensure the correct clamping of the µPADs. Square-shaped PADs of 20 × 20 mm were chosen. However, the samples’ shape is irrelevant since the holder can be modified without significantly affecting the operational functions of the instrument. For the possibility of easy removal and multiple analyses, a multi plates PAD-holder disc was also created; as a result, the system was required to constrain each plate in the correct position to ensure quick positioning and disconnection.

The darkroom panel was another fundamental component since the instrument must have a darkroom, which would facilitate the correct and repeatable reading of the samples, and at the same time, guarantee the correct assembly and isolation of the electronic elements. This part is also the structural component for the pin that supports the PAD-holder.

Some gears were also constructed; they were necessary for the proper operation of the instrument, which has the task of rotating the sample-holder 360°. Additionally, in this case, they were sized based on the needed dimensions and gear ratio and as the forces involved were weak, it was possible to create the gears using plastic material (PLA) and a simple 3D printer.

PLA+ was employed since it is a material that lends itself very well to rapid prototyping and 3D printing with excellent results in terms of manufacturing quality, mechanical properties, low cost, and workability (due to its relatively low melting temperature of 200 °C). In addition, this material is non-toxic, can be recycled quite well, and demonstrated excellent chemical resistance to the materials used for our samples. Furthermore, using 3D printing, with molten material deposition technology (FDM), it was possible to prototype all the components in a highly detailed way and immediately ensure they were ready for final assembly without any other machining. Moreover, thanks to the 3D modeling of the components, it was possible to move from the virtual 3D model to the actual physical model in a short time.

**Figure 1:**
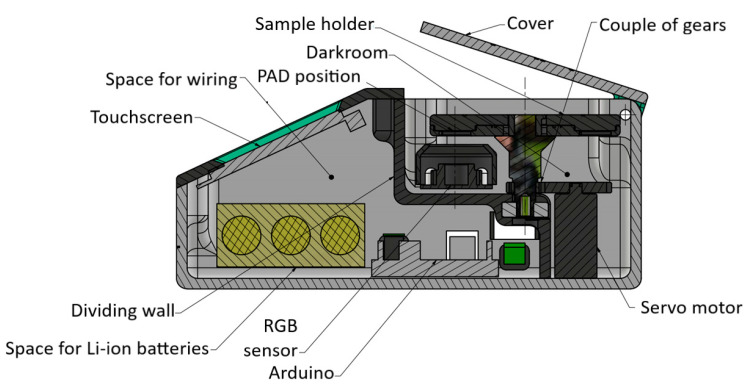
RGB-detector’s placement scheme.

Figure 1 shows the RGB-detector’s placement scheme.

The RGB-detector’s code was written by Arduino IDE software 1.8.13. The code used the following libraries: Nextion.h, dedicated to the touchscreen; Servo.h, for the servomotor control; math.h, which includes several useful mathematical functions for manipulating floating-point numbers; and the sensor-specific library Panjkrc_tcs3200. The code also used Arduino’s required setup() and loop() functions.

### 3.3. Calibration of the RGB-Detector

The core of the RGB-detector is the TCS3200 sensor, i.e., a color light-to-frequency converter, which provides a square wave with the frequency output directly proportional to the light intensity.

**Figure 2:**
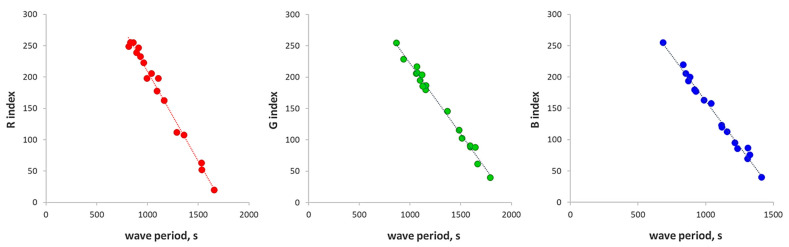
RGB-detector calibration: wave period vs. R. G and B indexes of colored cards.

A calibration was performed to correlate the wave period (T) and the RGB indexes. To this aim, colored cards at known RGB values were employed, and for each index, a linear relationship between the wave period and the index value was obtained, as shown in Figure 2.

The following three linear equations were so employed to convert the wave period to the corresponding RGB values (the number in parenthesis is the uncertainty for the last digit expressed as the standard error of the parameter obtained by the linear regression of the data):
R = −0.287(8) × T + 496(9)R^2^ = 0.987G = −4.4(1) × T + 1978(18)R^2^ = 0.990B = −3.41(9) × T + 1552(15)R^2^ = 0.988

### 3.4. RGB-Detector Application: Green µPADs Array for pH Measurements

The ability of the RGB-detector to quantitatively measure the color of µPADs was demonstrated by applying it to an array of green µPADs developed for pH measurements.

**Figure 3:**
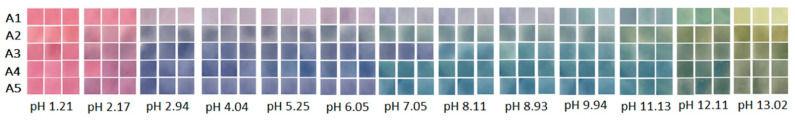
green µPADs array for pH measurement. A1 = 100%RC; A2 = 75%RC-25%BPF; A3 = 50%RC-50%BPF; A4 = 25%RC-75%BPF; A5 = 100% BPF. Three replicates for each pH value.

As mentioned, the array was prepared with five kinds of µPADs obtained by drop-coating filter paper’s square cutouts with aqueous extracts of pigments from red cabbage (Brassica oleracea, RC) and butterfly pea flower (Clitoria ternatea, BPF). The µPADs of the array expressed different colors when in contact with aqueous solutions at pHs ranging from 1 to 13. A picture of the array is provided in Figure 3.

Using the RGB-detector, the color indexes of each µPAD were acquired, and the RGB matrix of data, which was adequately organized, was submitted for multivariate analysis.

The first tools applied were 3WPCA and PCA for the preliminary data rationalization, visualization and pattern recognition.

**Figure 4:**
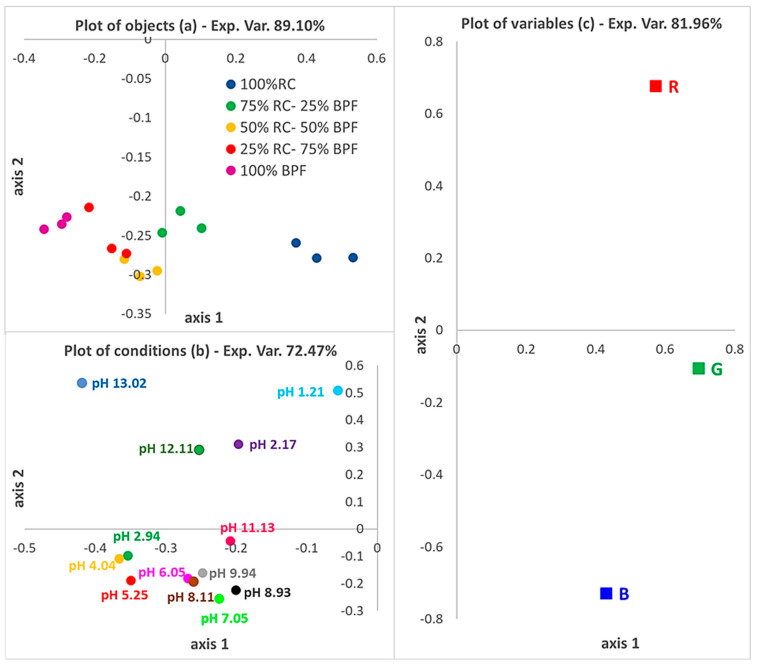
The loading plots of the 3−Way PCA model on the first two axes: Plot of the objects (**a**), Plot of conditions (**b**) and Plot of variables (**c**). Exp. Var. = cumulative % variance explained after unfolding.

Figure 4 shows the 3WPCA plots; in each of them, the loading values of the three modes (objects, conditions and variables) are reported. The five types of µPADs obtained with five different extracts (objects) show loading values arranged along the horizontal axis (axis 1) due to the different brightness, which increases with the decrease in the percentage of BPF and the corresponding increase in the percentage of RC. This assumption is confirmed by the loadings of the R, G, and B indexes (variables) as they all have a positive value on the *x*-axis, which indicates that their values increase, moving from the left to the right of the plot, and leading to brighter colors by increasing the percentage of RC. Conversely, on the *y*-axis, R has a positive loading value while G and B have negative values, suggesting that by the change of the pH, the R index increase while the G and B index decreases, which corresponds to the numerical effect of the clear colors’ variations for the µPADs array in contact with solutions at the extremes of the pH range. Regarding the pHs (conditions), there is a clear distinction between pH < 4 and pH > 9 that is easily recognizable, as well as in both the differences in the color shade (separation along axis 2) and the brightness (separation along axis 1); conversely, for a pH between 5 and 8, the difference is smaller as expected since there is a similar color of the PADs in this pH range.

**Figure 5:**
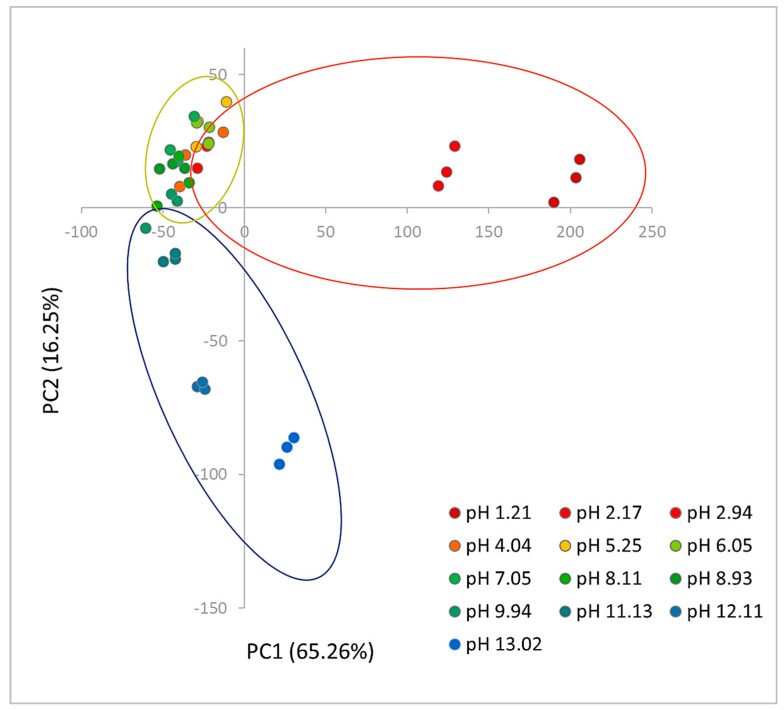
The score plot of the PCA model on the first two principal components. The ellipsoids are added to identify the clusters at the different pH subintervals and later used for building the PLS models.

PCA was also applied to assess if there were clusters of data. The model was obtained considering the first two components, which explain 81.51% of the experimental variance (65.26% PC1 and 16.25% PC2). The PCA score plot is shown in Figure 5. It can be observed that the samples were grouped into the following three clusters: a first one (red ellipsoid) for samples at pH < 5, a second cluster (little green ellipsoid) highlighted for samples at a pH between 5 and 8 and the third (blue ellipsoid) for samples at a pH from 9 to 13.

After identifying the three pH subintervals, partial least squares regression (PLS) was applied. Three PLS models were developed for pH 1 ÷ 4, B for pH 5 ÷ 8 and C for pH 9 ÷ 13. The models were validated by predicting the test samples and comparing the values obtained with those measured experimentally with a pH-meter.

Table 1 reports the number of components used to develop the PLS models, the percentage of explained variance in cross-validation (% Exp. Var. CV), and the root mean square error in CV (RMSECV).

The minimum RMSECV is obtained with five components for models B and C, and with three for model A. The explained variance % is high in all cases, i.e., higher than 92%. The RMSECV values are low, indicating high precision and good agreement with the values measured by the pH meter. Interestingly, the RMSECV for model A is similar to that achievable with glass electrodes at pH values of lower than 2.

**Figure 6:**
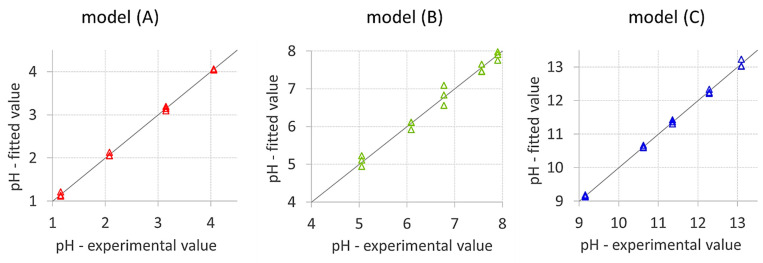
PLS experimental vs. fitted plots for models **A** (red triangles), **B** (green triangles) and **C** (blue triangles). RGB indexes were acquired by the RGB-detector.

Figure 6 shows the experimental vs. fitted data graphs for each model; the validation test results are reported in Table 2.

The PLS models developed proved their abilities in predicting the pH of all samples; this means that the pH values obtained from the color indexes of the green µPADs array acquired by the RGB-detector are accurate; indeed, the relative error % is always less than 5%. Moreover, the data precision is comparable to that of standard pH-meters.

## 4. Conclusions

This work reports the design, realization, and example of the application of a user-friendly, low-cost detector that is able to read the RGB indexes of green microfluidic paper-based analytical devices (µPADs).

The device, named the RGB-detector, is an accessible option for color measurement using spectrophotometers, smartphones, or tablets, whose drawbacks are the sensitivity variation among different devices and the need for image processing to acquire RGB values, making real-time monitoring time consuming.

The ability of the RGB-detector to read the color of micro-fluidic paper-based analytical devices (µPADs) is demonstrated by applying it to an array of green µPADs for pH measurements as a proof-of-concept. The RGB indexes extracted were submitted for chemometric data treatment and good results were obtained in terms of precision and agreement with the pH values measured with a classical pH-meter.

The good performances obtained makes the RGB-detector promising for application to microfluidic devices based on substrates other than paper. Furthermore, the possibility of 3D printing being employed to construct interchangeable sample-holders for microfluidic devices of different shapes and dimensions is an added value for expanding the applicability of the RGB detector prototype realized here.

## Figures and Tables

**Table 1:** The number of components, % explained variance in cross-validation (%Exp.Var.CV), and root mean square error in CV (RMSECV) for PLS models A, B and C.

Model	n. comp.	%Exp.Var. CV	RMSECV
**A**	3	99.82	0.0537
**B**	5	92.08	0.2892
**C**	5	98.65	0.1708

**Table 2:** pH values of samples used as a test set for validation of PLS models. Comparison between the values measured by a pH-meter and those obtained by the green µPADs array-PLS models. RGB indexes were acquired with the RGB-detector.

Sample	pH (pH-Meter)	pH (Green µPADs Array) ^1^	Relative Error %
Schweppes	2.39	2.35(3)	1.7
Sprite	2.72	2.84(1)	4.2
White wine vinegar	3.05	3.03(2)	0.6
Tropical aloe vera	3.55	3.51(1)	1.1
Tap water	7.68	7.77(6)	1.2
Ammonia cleaner	10.73	10.80(3)	0.7

^1^ Data are reported as the mean value of three replicates. The number in parenthesis is the uncertainty on the last digit expressed as standard deviation.

## Data Availability

Not applicable.
